# Prevalence of Body Area Work-Related Musculoskeletal Disorders among Healthcare Professionals: A Systematic Review

**DOI:** 10.3390/ijerph20010841

**Published:** 2023-01-02

**Authors:** Julien Jacquier-Bret, Philippe Gorce

**Affiliations:** International Institute of Biomechanics and Occupational Ergonomics, Université de Toulon, CS60584, CEDEX 9, 83041 Toulon, France

**Keywords:** musculoskeletal disorders, prevalence, body area, health professionals, nurses, dentists, physiotherapists, osteopaths, surgeons, midwives, risk factors, response and treatment, worldwide analysis

## Abstract

Healthcare professionals perform daily activities that can lead to musculoskeletal disorders (MSDs). The objective of this review was to summarize these MSDs by body areas in relation to healthcare professions. The underlying question is, worldwide, whether there are areas that are more exposed depending on the occupation or whether there are common areas that are highly exposed to MSDs. This issue has been extended to risk factors and responses to reduce MSDs. The review was conducted according to the PRISMA guidelines between February and May 2022. Google scholar and Science Direct databases were scanned to identify relevant studies. Two authors independently reviewed, critically appraised, and extracted data from these studies. Overall and body area prevalence, risk factors, and responses to MSDs were synthetized by occupational activity. Among the 21,766 records identified, 36 covering six healthcare professions were included. The lower back, neck, shoulder and hand/wrist were the most exposed areas for all healthcare professionals. Surgeons and dentists presented the highest prevalence of lower back (>60%), shoulder and upper extremity (35–55%) MSDs. The highest prevalence of MSDs in the lower limbs was found for nurses (>25%). The main causes reported for all healthcare professionals were maintenance and repetition of awkward postures, and the main responses were to modify these postures. Trends by continent seem to emerge regarding the prevalence of MSDs by healthcare profession. Africa and Europe showed prevalence three times higher than Asia and America for lower back MSDs among physiotherapists. African and Asian nurses presented rates three times higher for elbow MSDs than Oceanians. It becomes necessary to objectively evaluate postures and their level of risk using ergonomic tools, as well as to adapt the work environment to reduce exposure to MSDs with regard to the specificities of each profession.

## 1. Introduction

Musculoskeletal disorders (MSDs) are very common among healthcare professionals. The prevalence of MSDs among several healthcare professions was investigated. Prevalence of over 80% has been reported among physiotherapists [1], masseurs [2], nurses [3], midwives [4], dentists [5] and surgeons [6]. The high exposure to MSDs is directly related to their practice, which requires varied tasks and a high physical load. Numerous studies have highlighted the use of repeated awkward postures that are often static, particularly among surgeons [7] and physiotherapists [8].

Patient handling or transfers have also been observed in nurses [9] and physiotherapists [10]. Accuracy is also a factor in the origin of MSDs, as has been shown in dentists [11] and surgeons [12].

Understanding the mechanisms that lead to the appearance of MSDs requires knowledge of the most exposed body areas. A large number of studies have reported that the lower back was a highly exposed area in physiotherapists [13], nurses [14], and surgeons [15]. Neck and shoulder have also been reported as exposed areas in healthcare professionals [8,16,17,18,19]. More specific studies, such as on the thumbs of masseurs [20,21], have also been carried out to evaluate the prevalence of MSDs. Risk factors and responses/treatment implemented to reduce symptoms have also been used to further study MSDs in healthcare professionals. Muaidi and Shanb [22] reported this information for physiotherapists, Tinubu et al. [23] for nurses, and Mohseni-Bandpei et al. [24] for surgeons.

In the literature, studies have presented syntheses through reviews that essentially reported the prevalence of MSDs in a given occupation, such as the work of Vieira et al. on physiotherapists [25], Saberipour et al. on nurses [26], and Epstein et al. on surgeons [27]. These reviews make it possible to draw conclusions about the measures to be implemented to reduce the impact of MSDs. These works were carried out either for a limited number of zones, a single country or by occupation. However, due to the importance of the MSD issue for health professionals, it would be interesting to summarize the prevalence by body area, including all health professions for which information is available worldwide. This review would provide a global view to better understand the MSD issue by taking into account healthcare professions and if there could be a trend induced by the continents in which the work has been carried out. The objective was to describe the prevalence of MSD for different body areas in different health professions and to assess potential differences. The underlying questions were: (1) Are there specific zones for each profession, or are there common zones that are highly exposed to MSDs? (2) Are there specific factors of risk and response to MSDs in relation to each occupational activity?

## 2. Materials and Methods

This study was reported according to PRISMA guidelines for reporting systematic reviews and meta-analyses [28]. It was conducted between February 2022 and May 2022.

### 2.1. Search Strategy and Eligibility Criteria

The search strategy was applied to Science Direct and Google Scholar databases. The following keywords were used: “Musculoskeletal disorders” AND “Healthcare professional” AND “Body area”. The search focused exclusively on English language peer-reviewed works that quantified the MSD prevalence by body area in healthcare professionals. The search was limited to articles published between 2000 and 2022. Reviews, systematic reviews, commentaries, case studies and case series were not retained. Articles were included if they were original research that studied the prevalence of work-related MSDs among healthcare professionals without any restriction. The search focused on work that addressed the prevalence of MSDs by body area (neck, back, upper and lower limbs). Studies were excluded if they:

- were not published in English,
- were not about healthcare professionals,
- mixed healthcare professions without the possibility of distinguishing between them,
- provided insufficient work-related MSD details,
- provided insufficient data about sampling,
- excluded or focused on only a limited number of body areas.

Results were imported from both databases and compiled to remove duplicates. Two reviewers (PG and JJB) separately screened all titles and abstracts of unique records for eligibility. Full-text manuscripts of all relevant titles/abstracts were obtained, and the relevance of each study was assessed according to the inclusion/exclusion criteria by the two reviewers separately. Studies that did not meet the criteria were excluded. All discrepancies were resolved by consensus and re-review of the articles.

### 2.2. Methodological Quality Assessment and Risk of Bias

The quality of the included articles was assessed independently by two reviewers using the modified CONSORT 2010 checklist (Table A1) [29]. The presence of each item (where applicable) was checked, and the evaluation obtained by each reviewer was compared. The discrepancies were discussed for the final evaluation, involving a third reviewer where necessary. The quality appraisal was obtained using McFarland and Fischer [30] classification:

- at least 85% of the checklist items are high-quality articles,
- less than 50% of the checklist items are low-quality articles,
- otherwise, they are considered of medium quality.

### 2.3. Data Extraction

The following data were extracted from the included articles: number of participants, healthcare profession, response rate (survey), male and female distribution, age, country, overall MSD prevalence and MSD prevalence by body area. Any element related to work-related MSDs such as risk factors, their impact on work habits or the strategies used to respond to and treat them were also considered. Based on the information collected in each study, a synthesis was proposed by healthcare profession.

## 3. Results

### 3.1. Search Results

**Figure 1:**
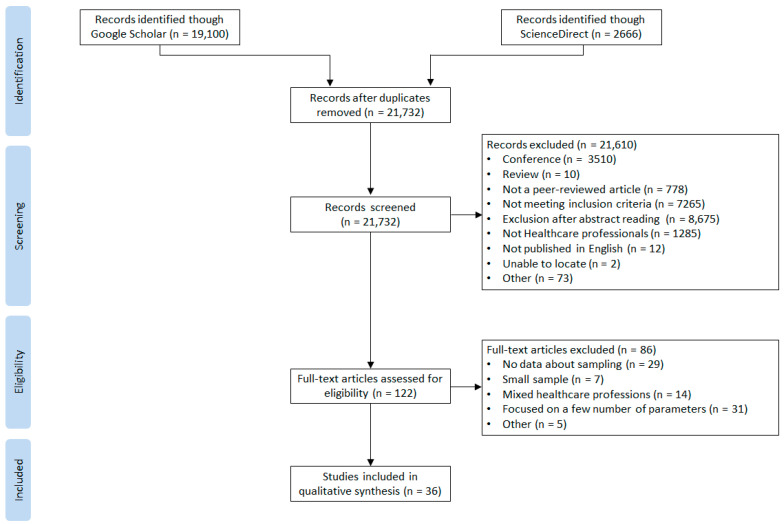
PRISMA Flow Chart.

The searches identified 21,766 records. After duplicates were removed, 21,732 articles remained, and 21,610 were excluded from the title/abstract screening. Among the 122 remaining articles, 86 were excluded after full reading because either the data were mixed and did not meet the objective or the parameters studied were insufficient. Thirty-six articles were finally included in the present review. The search process is shown in Figure 1.

### 3.2. Quality Appraisal

The quality appraisal of the 36 included articles revealed that 34 studies were of average quality (percentage of items present between 50 and 85%). The studies by Glover et al. [8] and Attar [31] were considered of high quality with 87% and 89% of items present, respectively (Table 1).

### 3.3. Study Characteristics

All included articles were surveys based on questionnaires about healthcare professionals. Among the 36 included studies, six healthcare professions were identified. Professionals were dentists or dentist hygienists (8 studies), midwives (2 studies), nurses (11 studies), osteopaths (1 study), physiotherapists or physical therapists (10 studies), and surgeons (4 studies). Subjects were generally male or female aged between 20 and 55 years. Three studies focused on nurses were conducted with only females [18,51,55]. Two studies did not provide information about gender [5,9]. The samples in the different studies were heterogeneous, ranging from 32 surgeons [44] to 2688 physiotherapists [8].

The selected studies covered a wide range of countries from different continents. Participants mainly came from public, private and university hospitals.

Table 2 summarizes the objectives, the health profession and the general population characteristics, and the prevalence of MSD by body area of the 36 included studies. Ten areas were identified. Neck and shoulder MSDs were addressed in all 36 of the included studies. All studies also evaluated back MSD prevalence. However, the descriptions differed between studies. Most of them focused on the lower back (31 studies). Information about the upper back (27 studies) or the mid back (8 studies) was also available in several studies. Elbow/forearm and wrist/hand/finger areas were assessed in 34 studies, and the lower limb joints, i.e., hips/thighs, knees, and ankles/feet were covered in 32 studies.

### 3.4. Body Area Work-Related MSD Prevalence

**Figure 2:**
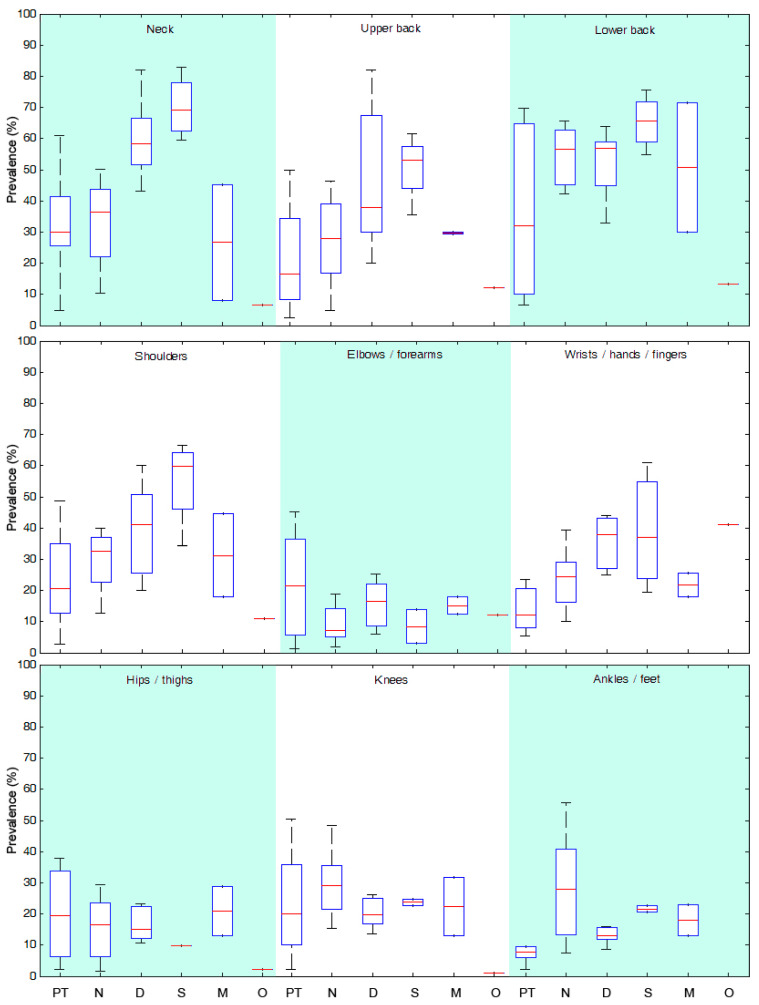
Synthesis of MSD prevalence by body area for each healthcare profession. Boxplots represent lower, median and upper quartile values. Whiskers correspond to the most extreme values within 1.5 times the interquartile range. PT = Physiotherapists/Physical therapists; N = Nurses; D = Dentists; S = Surgeons; M = Midwives; O = Osteopaths.

**Figure 3:**
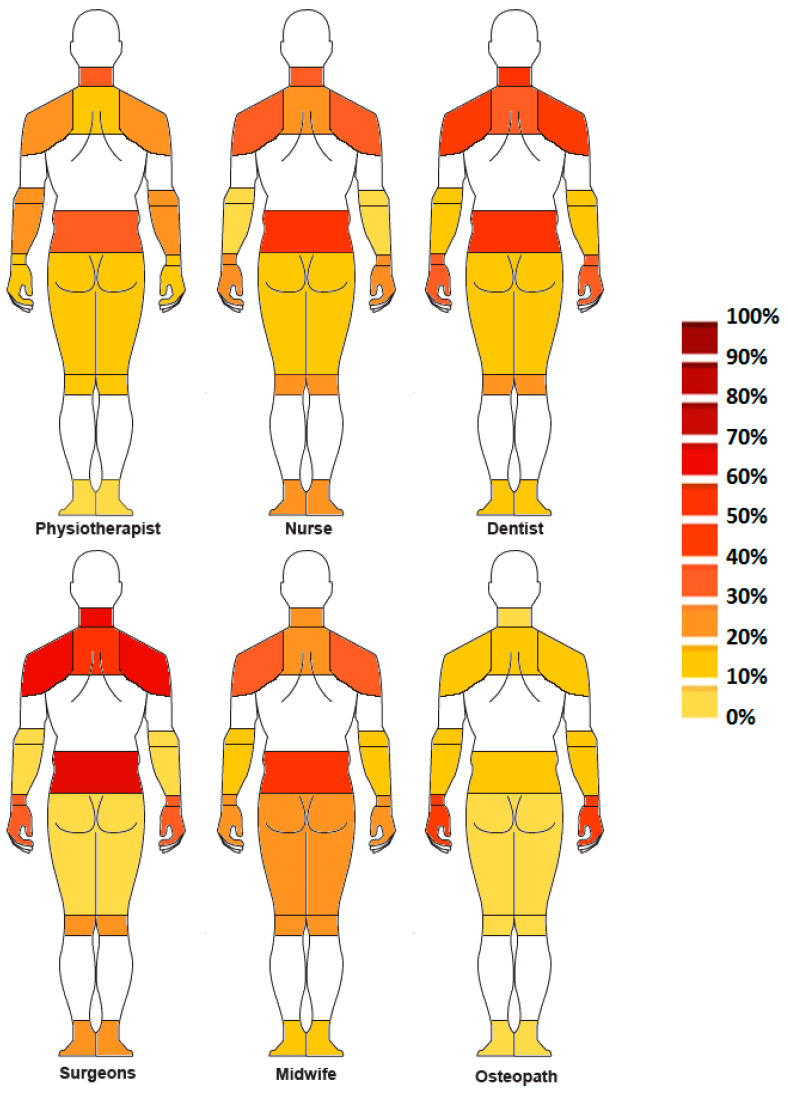
Body mapping of MSD prevalence by body areas and by healthcare profession. PT = Physiotherapists/Physical therapists.

Figure 2 and Figure 3 summarize the work-related MSD prevalence by body area and occupation. Across all healthcare professions, the neck and lower back were the most exposed areas, with a high average prevalence ranging from 26.7% to 70.1%. For the neck, dentists and surgeons were the two professions with the highest prevalence (above 60%), with maximum values of over 80% [42,52]. Physiotherapists, nurses and midwives presented a lower average prevalence, of 32.0%, 33.1% and 26.7%, respectively, but with a significant range of 37–55% [4,18,38,47,54].

About the lower back, MSD prevalence was higher than 50% for nurses, dentists, surgeons, and midwives, with maximum values of 65.7% [31], 64.0% [42], 75.6% [32], and 71.4% [47] respectively. Physiotherapists had a lower average rate (36.5%) but with a large range that highlighted a large disparity in the results proposed in the literature, with values ranging from 6.6% [38] to 69.8% [33].

The least exposed areas were the elbow/forearm and the lower limb joints, i.e., hip/thigh, knee/leg and ankle/foot, with a mean MSD prevalence of 14.9%, 17.8%, 25.0% and 20.0%, respectively.

The shoulder and wrist were differentially exposed depending on the profession. Dentists and surgeons demonstrated the highest prevalence, of 55.1% and 39.4%, respectively, for the shoulder and 39.1% and 38.8%, respectively, for the wrist, with maximum values above 60% [5,32]. For physiotherapists, nurses and midwives, the average rates were between 15.7% and 31.3%.

In four high-risk areas, i.e., neck, lower back, shoulders and wrists, surgeons and dentists appeared to be the healthcare professionals most exposed to MSDs, and physiotherapists, midwives, and nurses to a lesser extent. Nurses were the professionals whose lower limbs were the most exposed, with an average prevalence of over 25%, compared with 18% for the others.

The study on osteopaths showed that the wrist was the most exposed area, with MSD risks that were the highest (41.1%) in comparison to the other healthcare professions [46].

Regardless of the affected areas, Table 3 summarizes the overall MSD prevalence for 26 of the 36 studies included in the review. Nurses, midwives, dentists and surgeons demonstrated prevalence above 80%. Physiotherapists had an average prevalence of 55%, but with a wide range. Four studies ([10,17,33,54]) reported rates above 80% for the other health professions listed above, while three others ([22,34,38]) evidenced significantly lower MSD prevalence (<50%).

Table 4 presents the MSD prevalence by body area and healthcare profession in relation to each continent. Concerning physiotherapists, Africa (20.6%) and Europe (24.4%) showed prevalence rates twice as high as those for the other continents for wrist/hands (10.2 to 13.2%) and at least three times higher than Asia and America for lower back (69.8% and 62.9% vs. 21.6% and 6.6%, respectively). America (66.0%) and Oceania (62.5%) presented prevalence rates two times higher than those for Asia (39.8%) and Europe (37.2%) for mid back MSDs, while Oceania (41.0%) and Europe (33.5%) presented prevalence rates two times higher compared to the other three continents for shoulder (Africa 22.2%, Asia 12.7%, and America 19.1%). Finally, Africa (5.6% for elbow and 6.3% for hip/thigh) had three to four times lower prevalence than other continents for elbow and hip/thigh, while Europe had the highest rates for the lower limbs.

Concerning nurses, Europe (neck: 50.1%, upper back: 40.9%) had a prevalence 1.5 times higher than that of the other continents for neck (30.3% to 35.5%) and upper back (11.3% to 27.4%). Africa (11.3%) and Asia (9.5%) presented rates three times higher for elbow than Oceania (3.2%), which also had prevalence twice as low as the other continents for ankle/feet (11.3% compared to 24.2–32.6%).

Finally, among dentists, America (60.0%) had the highest prevalence of shoulder MSDs compared to the other continents (from 26.0% for Asia to 48.9% for Oceania). Asia (82.0%) and America (67.4%) had prevalence two times higher than that of Europe (20.0%) and Oceania (35.2%) for the upper back.

### 3.5. Job Risk Factors

Ten articles on four of the health professions—two for nurses [9,23], one for osteopaths [43], five for physiotherapists [8,10,22,33,35], and two for surgeons [6,52]—associated risk factors with MSDs (Table A2). No work on midwives and dentists included in the review addressed this aspect. Eighteen risk factors common to all healthcare professions were identified. Seventeen of them were mentioned in at least six of the studies that addressed risk factors among the different health professions. Nine had a reported rate of over 50% and were listed in the majority of studies (7–8 of the 10 studies). “Working in a same position for a long time”, “Working in an Awkward/Cramped Position”, “Working when physically fatigued/in an injured state”, and “Performing the same task over and over” were the most reported factors in the literature (in nine of 10 studies) with significant average prevalence rates of 62.5%, 61.2%, 51.6%, and 56.0%, respectively. “Treating a large number of patients in a 1 day” reported in six studies was the factor with the highest prevalence (65.9%).

### 3.6. Responses and Treatment to Reduce the Symptoms of MSDs

Eleven articles related to all professions except dentists, including two for nurses [9,23], one for midwives [4], one for osteopaths [46], six for physiotherapists [8,10,17,22,33,35], and one for surgeons [6], reported a total of 21 responses/treatments used to reduce MSD symptoms (Table A3). “Modify patient’s position/my position”, “select techniques/procedure that will not cause or aggravate discomfort”, and “pause regularly to stretch and change posture” were the three most reported responses in the majority of works (8–9 of 11 studies). These were performed, respectively, by 54%, 52% and 38% of the practitioners. Four criteria were also cited in half of the studies for the majority of professions: “I use other body part in order to perform manual treatment/nurse procedure”, “I adjust plinth/bed height prior to the treatment of a patient”, “I warm up and stretch before performing my work manual techniques, nurse duties”, and “I stop a treatment if it causes or aggravates my discomfort”, with reported frequencies of 53%, 58%, 30%, and 48%, respectively. “I get someone else to help me handle a heavy patient” had the highest rate (67%) but was only reported by physiotherapists [17,22,33] and nurses [9,23].

## 4. Discussion

The aim of this study was to identify the prevalence of MSDs among healthcare professionals and to determine whether all were affected in the same way or whether specific areas were more exposed depending on the profession. Thirty-six studies were included in the analysis, covering six healthcare professions from different countries: nurses, midwives, physiotherapists, osteopaths, dentists, and surgeons.

The general prevalence showed very high rates of MSDs in all health professions with values above 75% for the majority of the jobs considered. Four body areas, i.e., the neck, the back (mainly the lower back but in some cases also the upper back), the shoulders and the upper extremities (wrists, hands, fingers), were widely exposed to MSDs, with significant prevalence for all of the different jobs. The neck area and back were widely considered in the different studies [4,16,47,56,57,58,59]. The results of these numerous works showed that regardless of the profession, the MSD prevalence rates were high [46,60,61].

This was mainly due to the awkward postures adopted by the professionals. Among nurses and physiotherapists, handling or transferring heavy materials/patients and prolonged static postures were the predominant situations [62,63,64].

Shoulders and extremities also showed significant rates, particularly among dentists and surgeons. This can be explained by the precision required and the constraints related to the interventions, such as unique accesses (to the mouth in particular) and the risks incurred when handling tools [11,12]. Physiotherapists and nurses perform many manual therapies or wound care procedures that place greater demands on the wrists and hands [21,50].

The least exposed areas were located in the lower limbs for all occupations, ranging from 15 to 25%. Nurses, however, had higher rates of MSD prevalence than other occupations. This result is in agreement with Reed’s work on the prevalence of lower limb MSDs in nurses [20]. This is related to the fact that their daily practice involves sequences of many static postures and many movements with many manipulations [50,65].

Numerous studies including risk factors have been carried out in particular among physiotherapists and nurses, as reported in this review (Table A2). A list of 17 common items was documented for the different included studies (including five for physiotherapists and two for nurses). “Working in a same position for a long time”, “Working in an Awkward/Cramped Position”, “Working when physically fatigued/in an injured state”, and “Performing the same task over and over” were the factors most reported in the literature on all professions (9/10 articles), with significant average prevalence rates of 62. 5%, 61.2%, 51.6%, and 56.0%, respectively, consistent with the results of studies on these different healthcare professions [7,66,67].

The review highlighted that these aspects were little considered in dentists, for whom no risk factors were identified in the eight articles included. These aspects were also little addressed in surgeons, who mainly reported risk factors related to workload such as lack of breaks and perseverance in work despite fatigue or injury, which were also found in other professions [6,52].

Healthcare professionals reported several responses to the presence of MSDs to reduce symptoms. “Modify patient’s position/my position,” “select techniques/procedure that will not cause or aggravate discomfort,” and “pause regularly to stretch and change posture” were the three most articulated responses regardless of occupation. Physiotherapists were the healthcare professionals with the most information, with numerous works and considered items (19 items) [66,68,69]. Nurses addressed these aspects to a lesser extent, with two studies and nine items [9,23]. For the other professions, this problem was studied to a limited extent, or not studied at all.

This literature review showed that healthcare professions involving specific tasks, such as dentists and surgeons, were the most exposed to MSDs, particularly in the neck, back, shoulders and wrists/hands. Occupations with more displacements, such as nurses and physiotherapists, presented lower but significant risks, with more exposed areas due to the heterogeneity of their activities. In particular, the risk of MSDs in the lower limbs was higher for nurses who walk a lot.

More generally, for both the risk factors and the solutions proposed to reduce MSDs, the redundant element that appeared, whatever the profession or continent, was posture. The daily activities performed in uncomfortable postures, repeated and maintained over time, are the cause of MSD risks. These risk factors must be analyzed by respecting general ergonomic principles such as adopting postures close to joint neutrality in order to reduce joint and muscle constraints.

In this context, ergonomic tools such as RULA [70], LUBA [71] and REBA [72] have been developed to quantify the risk of MSDs associated with a posture and thus evaluate the need to make changes in a given situation. They take into account the distance from the neutral position of the joint angles, for which the risk of MSDs is known to increase considerably, as well as handling of heavy loads, static postures or repetition of the same movement. These assessments allow us to objectively identify the areas at risk. Recent work among physiotherapists has shown that significant flexion of the neck and lower back, as well as significant flexion and abduction of the shoulders during massage, make these areas particularly exposed to MSD risks [73]. This result is consistent with the results proposed in this review and, therefore, appears to be generalizable to all health care professions.

These quantitative evaluations in healthcare professionals are very rarely performed. This is an approach that should be developed in order to reduce the risks of MSD occurrence [74].

At the same time, the working environment of healthcare professionals should be analyzed. Many devices are used to care for their patients. All practitioners work mainly in a static position, standing or sitting on stools, and often use treatment tables or beds to perform their interventions (operations, massages, manipulation, care, etc.) [75]. The adjustment of these devices, such as table heights, patient or screen positioning, are factors that could affect posture and, therefore, the MSD risk, particularly by increasing flexion and rotation. This was particularly apparent among professionals who reported that they change their position or their patient’s position in response to MSDs in different professions.

### Limitations

Some limitations should be addressed. The first concerns the method used in the different included studies. Indeed, the questionnaires proposed were not all the same, even though the common objective was to assess the prevalence of MSDs in the healthcare professions. These differences could lead to variability in the responses and cause MSD prevalence rates to vary. Differences in rates may also arise depending on how the responses are considered. Reporting responses of the entire sample or only of those who reported MSDs (thus excluding those who were healthy) could significantly alter the prevalence of MSDs. A harmonization of the survey methodologies conducted in the different countries and for the different professions would strengthen the present results.

A second limitation concerns the populations studied. Independently of the different healthcare professions considered, the nature of the respondents may also influence the results. Indeed, age, gender, status and years of experience (e.g., students, trainees versus experienced workers), and place of practice (private or hospital practice) are all factors to be considered when assessing the prevalence of MSDs. Inference by continent is only a tendency that must be limited due to the small number of studies for certain areas (lower limbs in particular) and professions (osteopaths and midwives). The analysis could not be carried out for risk factors and responses to reduce MSDs due to the small number of studies that addressed this issue. For the large majority, only one or two studies per continent were identified (Table A2 and Table A3).

Another limitation concerns the PRISMA selection method. First, the inclusion criteria for the articles led to the exclusion or potential omission of works that could have completed and supported the results of this literature review. Secondly, the choice of the three coupled keywords without using synonyms or multiples terms using AND/OR could have excluded, in spite of the more than 21,700 found, some relevant works with regard to the objective.

## 5. Conclusions

All healthcare professions are significantly exposed to MSDs. Four areas common to all professions are highly exposed: back, neck, shoulder, and hand/wrist. Some professions have areas more specifically affected according to their specificity, such as the shoulder and upper extremities for surgeons and dentists (35–55%) or the lower limbs for nurses (>25%). Surgeons and dentists presented the highest prevalence of lower back MSDs (>60%). The main causes reported for all health professionals are related to maintaining and repeating awkward postures. It is important to assess postures and associated MSD risks in various practices using ergonomic tools to identify the most exposed joints and body areas. Future works could be focused on work environment design, particularly the positioning and adjustment of equipment, and on postural analysis to reduce the occurrence of MSDs.

## Figures and Tables

**Table 1:** Quality appraisal of the included studies according to the modified CONSORT 2010 checklist.

High Quality	Medium Quality		Low Quality
Attar [31]	Adams et al. [32]	Adegoke et al. [33]	-
Glover et al. [8]	Alrowayeh et al. [34]	Anap et al. [9]	
	Anton et al. [5]	Anyfantis and Biska [35]	
	Asghari et al. [36]	Ayers et al. [37]	
	Campo et al. [38]	Choobineh et al. [39]	
	Chung et al. [10]	Cromie et al. [17]	
	Hayes et al. [40]	Jang et al. [41]	
	Kee and Seo [18]	Khan and Yee Chew [42]	
	Kierklo et al. [43]	Knudsen et al. [44]	
	Leggat and Smith [45]	Liang et al. [6]	
	McLeod et al. [46]	Muaidi and Shanb [22]	
	Munabi et al. [3]	Okuyucu et al. [4]	
	Okuyucu et al. [47]	Pugh et al. [48]	
	Rabiei et al. [49]	Ribeiro et al. [50]	
	Smith et al. [51]	Szeto et al. [52]	
	Szymańska [53]	Tinubu et al. [23]	
	Vieira et al. [54]	Yeung et al. [55]	

**Table 2:** Objectives and characteristics of the 36 included studies by healthcare profession. MSD prevalence by body area was reported for each study (when available).

Autors	Objective	Study Details				Prevalence	Studied Parameters										
							Main Body Area										
							Neck	Upper Back	Mid Back	Lower Back	Shoulders	Elbows/Forearms	Wrists/Hands/Fingers	Hips/Thighs	Knees	Ankles/Feet	Whole Body
**Adegoke et al., 2008 [33]**	Investiagtion of MSD prevalence, risk factors, and treatment among Nigerian physiotherapists	**Population**	Physiotherapists	**Male/female**	63.5%/36.5%		31.1%	14.3%	-	69.8%	22.2%	5.6%	20.6%	6.3%	15.9%	9.5%	-
		**N-participant**	120	**Age (year, mean ± SD)**	33.7 ± 6.8												
		**Response rate**	58%	**Country**	Nigeria												
**Alrowayeh et al., 2010 [34]**	Investigation of MSD prevalence among physical therapists in the State of Kuwait	**Population**	Physical therapists	**Male/female**	53%/47%		20.2%	19.0%	-	32.0%	12.6%	3.7%	10.8%	3.3%	10.8%	6.1%	-
		**N-participant**	212	**Age (year, mean ± SD)**	36.5 ± 9.1												
		**Response rate**	63%	**Country**	State of Kuwait												
**Anyfantis et al., 2017 [35]**	Investiagtion of MSD prevalence and risk factors among Greek physiotherapists	**Population**	Physical therapists	**Male/female**	52.4%/47.6%		41.3%	49.8%	-	62.9%	48.6%	36.5%	43.3%	37.8%	42.9%	33.3%	-
		**N-participant**	252	**Age (year, mean ± SD)**	42.18 ± 9.21												
		**Response rate**	79.00%	**Country**	Greece												
**Campo et al., 2008 [38]**	Investigation of 1-year MSD prevalence and effects of risk factors in United States	**Population**	Physical therapists	**Male/female**	28.8%/71.2%		4.9%	2.4%	-	6.6%	3.2%	1.4%	5.3%	2.3%	2.1%	2.2%	20.7%
		**N-participant**	881	**Age (year, mean ± SD)**	40.3												
		**Response rate**	67.00%	**Country**	United States												
**Chung et al. 2013 [10]**	Investiagtion of MSD prevalence, risk factors, and treatment among Korean physical therapists	**Population**	Physiotherapists	**Male/female**	52.9%/47.1%		28.7%	-	53.5%	-	15.9%	45.2%	7.0%	33.8%	7.6%	8.9%	22.3%
		**N-participant**	157	**Age (year, mean ± SD)**	29.45 ± 4.14												
		**Response rate**	67.10%	**Country**	Korea												
**Cromie et al. 2000 [17]**	Investiagtion of MSDs prevalence, specialty areas, risk factors, and treatment among Australian therapists	**Population**	Physiotherapists	**Male/female**	22%/78%	12 month	47.6%	-	62.5%	-	41%	22.9%	13.2%	21.80%	33.60%	7.3%	11.2%
		**N-participant**	536	**Age (year, mean ± SD)**	38												
		**Response rate**	68%	**Country**	Australia												
**Glover et al. 2005 [8]**	Investiagtion of MSDs prevalence among physiotherapists, physiotherapy assistants and physiotherapy students in the UK	**Population**	Physiotherapists	**Male/female**	14%/86%	12 months	25.7%	-	37.2%	-	18.4%	14.8%	5.5%	13%	17.80%	4.8%	7.8%
		**N-participant**	2688	**Age (year, mean ± SD)**	39.50 ± 12.07												
		**Response rate**	73%	**Country**	UK	Career	33%	-	48%	-	23%	20%	8%	17%	23.00%	6%	10%
**Jang et al. 2006 [41]**	Investigation of 12-month MSD prevalence and risk factors among massage practitioners in Taiwan	**Population**	Massage therapists	**Male/female**	68.9%/31.1%	12 months	25.5%	-	19.3%	11.2%	19.3%	31.7%	23.6%	28.60%	50.30%	6.8%	13%
		**N-participant**	161	**Age (year, mean ± SD)**	37.7 ± 10.7												
		**Response rate**	82%	**Country**	Taiwan												
**Muaidi et al. 2016 [22]**	Investigation of MSD prevalence, causes, and impact among physical therapists in the Kingdom of Saudi Arabia	**Population**	Physiotherapists	**Male/female**	59.1%/40.9%	12 months	26.5%	-	46.5%	-	2.9%	12.2%	10.2%	16.40%	20.10%	8%	10.9%
		**N-participant**	690	**Age (year, mean ± SD)**	-												
		**Response rate**	69%	**Country**	Kingdom of Saudi Arabia												
**Vieira et al. 2015 [54]**	Investigation of MSD rates and characteritics among physical therapists according to their specialty and setting in United States	**Population**	Physiotherapists	**Male/female**	32%/68%	12 months	61%	-	66%	-	35%	42%	15%	36%	-	23%	36%
		**N-participant**	122	**Age (year, mean ± SD)**	43 ± 12												
		**Response rate**	n/a	**Country**	United States												
**Anap et al., 2013 [9]**	Investigation of MSD prevalence, job risk factors, and treatment among hospital nurses in India.	**Population**	Nurses	**Male/female**	n/a	12 months	31.1%	10.5%	-	48.2%	34.6%	1.9%	-	1.6%	29.0%	7.6%	81.0%
		**N-participant**	228	**Age (year, mean ± SD)**	31.4												
		**Response rate**	89.10%	**Country**	India												
**Asghari et al., 2019 [36]**	Investigation of MSD occurrence and risk factors among operator room nurses in Iran	**Population**	Nurses	**Male/female**	19.7%/80.3%	12 months	44.9%	33.2%	-	61.9%	33.3%	19.0%	31.3%	23.8%	60.5%	55.8%	92.5%
		**N-participant**	144	**Age (year, mean ± SD)**	34.6 ± 6.6												
		**Response rate**	100%	**Country**	Iran												
**Attar et al., 2014 [31]**	Investigation of MSD frequency and risk factors among nursing personnel in Saudi Arabia	**Population**	Nurses	**Male/female**	4.5%/95.5%	12 months	20.0%	-	5.0%	65.7%	29.0%	3.0%	10.0%	16.5%	21.0%	41.5%	-
		**N-participant**	200	**Age (year, mean ± SD)**	34.9 ± 8.1												
		**Response rate**	100.00%	**Country**	Saudi Arabia												
**Choobineh et al., 2006 [39]**	Investigation of MSD prevalence and relationship between perceived demands and reported MSDs among hospital nurses in Iran	**Population**	Nurses	**Male/female**	15.3%/84.7%	12 months	36.4%	46.4%	-	54.9%	39.8%	17.9%	39.3%	29.3%	48.4%	52.1%	-
		**N-participant**	641	**Age (year, mean ± SD)**	32.03 ± 8.02												
		**Response rate**	100%	**Country**	Iran												
**Kee and Sao, 2007 [18]**	Investigation of MSD prevalence based on intensity among Korean nurses	**Population**	Nurses	**Male/female**	0%/100%	12 months	17.3%	12.9%	-	23.4%	27.2%	7.4%	21.6%	9.9%	24.7%	17.3%	56.8%
		**N-participant**	162	**Age (year, mean ± SD)**	29.9 ± 6.3	12 months - Moderate	15.4%	10.5%	-	20.4%	25.3%	6.2%	17.9%	8.6%	22.8%	15.4%	53.7%
		**Response rate**	100%	**Country**	Korea	12 months - High	10.5%	4.9%	-	9.9%	17.3%	4.3%	11.7%	5.6%	15.4%	11.1%	45.7%
**Munabi et al., 2014 [3]**	Investiagtion of MSD prevalence and risk factors among nursing professionals in Uganda	**Population**	Nurses	**Male/female**	14.3%/85.7%	12 months	36.9%	35.8%	-	61.9%	32.6%	15.4%	29.1%	27.9%	37.1%	38.1%	80.8%
		**N-participant**	741	**Age (year, mean ± SD)**	35.4 ± 10.7												
		**Response rate**	85.40%	**Country**	Uganda												
**Pugh et al., 2020 [48]**	Investigation of MSD severity from pre-registration to 12-month registered nurses in Australia	**Population**	Nurses	**Male/female**	12%/88%	12 months	35.5%	11.3%	-	53.2%	32.3%	3.2%	16.1%	9.7%	17.7%	11.3%	-
		**N-participant**	111	**Age (year, mean ± SD)**	29.7 ± 11.2												
		**Response rate**	100%	**Country**	Australia	Career	44.0%	17.0%	-	63.0%	30.0%	7.0%	21.0%	12.0%	18.0%	28.0%	-
**Ribeiro et al., 2016 [50]**	Investigation of MSD nurses’ self-reported symptoms and risk factors in primary health care	**Population**	Nurses	**Male/female**	16%/84%	12 months	50.1%	40.9%	-	63.1%	37.8%	7.2%	28.4%	8.9%	25.2%	26.4%	-
		**N-participant**	409	**Age (year, mean ± SD)**	39.5 ± 8.8												
		**Response rate**	5.4%	**Country**	Portugal												
**Smith et al., 2004 [51]**	Investigation of MSD prevalence and risk factors among Chinese profesionnal nurses by department and body area	**Population**	Nurses	**Male/female**	0%/100%	12 months	42.8%	38.9%	-	56.7%	38.9%	10.0%	27.8%	22.8%	31.1%	34.4%	70.0%
		**N-participant**	180	**Age (year, mean ± SD)**	32.7 ± 7.9												
		**Response rate**	84.10%	**Country**	China												
**Tinubu et al., 2010 [23]**	Investiagtion of MSD prevalence, risk factors, and treatment among Nigerian nurses	**Population**	Nurses	**Male/female**	2.5%/97.5%	12 months	28.0%	16.8%	-	44.1%	12.6%	7.1%	16.2%	3.4%	22.4%	10.2%	-
		**N-participant**	118	**Age (year, mean ± SD)**	36.4 ± 7.7												
		**Response rate**	80%	**Country**	Nigeria												
**Yeung et al., 2005 [55]**	Investigation of MSD prevalence and risk factors among nurses in Hong Kong	**Population**	Nurses	**Male/female**	0%/100%	12 months	19.6%	22.7%	-	42.3%	20.6%	7.2%	17.5%	20.6%	29.9%	19.6%	-
		**N-participant**	97	**Age (year, mean ± SD)**	35.0 ± 7.0												
		**Response rate**	100%	**Country**	Hong Kong												
**Anton et al., 2002 [5]**	Investigation of carpal tunnel syndrom and other MSD pervalence among dental hygienists in United States	**Population**	Dental hygienists	**Male/female**	n/a	12-months	68.5%	67.4%	-	56.8%	60.0%	21.1%	69.5%	19.0%	13.7%	15.8%	-
		**N-participant**	95	**Age (year, mean ± SD)**	37.6 ± 7.9												
		**Response rate**	100.00%	**Country**	United States												
**Ayers et al., 2009 [37]**	Investigation of MSD prevalence and occupational health status of New Zealand dentists	**Population**	Dentists	**Male/female**	68%/32%	12-months	59.0%	30.0%	-	57.0%	45.0%	10.0%	25.0%	15.0%	21.0%	13.0%	-
		**N-participant**	566	**Age (year. mean ± SD)**	n/a												
		**Response rate**	77.00%	**Country**	New Zealand												
**Hayes et al., 2009 [40]**	Investigation of MSD prevalance among dental hygiene students in Australia	**Population**	Dental Hygienist	**Male/female**	5.6%/94.4%		64.3%	41.3%	-	57.9%	48.4%	7.1%	42.1%	11.9%	26.2%	12.7%	-
		**N-participant**	126	**Age (year, mean ± SD)**	26.4 ± 6.2												
		**Response rate**	71.60%	**Country**	Australia												
**Khan and Yee Chew, 2013 [42]**	Investigation of MSD prevalence among dental students in Malaysia	**Population**	Dental students	**Male/female**	26%/74%		82.0%		-	64.0%	26.0%	23.0%	42.0%	-	-	-	-
		**N-participant**	575	**Age (year, mean ± SD)**	n/a												
		**Response rate**	81.00%	**Country**	Malaysia												
**Kierklo et al., 2011 [43]**	Investigation of MSD symptoms and prevalence among dentists in northeast Poland	**Population**	Dentists	**Male/female**	11.8%/88.2%		47.0%	20.0%	-	32.9%	20.1%	15.1%	29.2%	23.3%	16.0%	15.0%	-
		**N-participant**	220	**Age (year, mean ± SD)**	n/a												
		**Response rate**	100.00%	**Country**	Poland												
**Leggat et al., 2006 [42]**	Investigation of MSD impact among dentists in Australia	**Population**	Dentists	**Male/female**	73.3%/26.7%	12-months	57.5%	34.4%	-	53.7%	53.3%	18.0%	33.7%	12.6%	18.9%	11.6%	-
		**N-participant**	283	**Age (year, mean ± SD)**	45.2 ± 11.9												
		**Response rate**	73.10%	**Country**	Australia												
**Rabiei et al., 2011 [49]**	Investigation of MSD prevalence among dentists in Iran	**Population**	Dentists	**Male/female**	64.1%/35.9%	12 months	43.0%	-	-	35.8%	25.0%	6.0%	25.0%	10.8%	19.6%	8.7%	-
		**N-participant**	92	**Age (year, mean ± SD)**	30.1 ± 8.7												
		**Response rate**	58.00%	**Country**	Iran												
**Szymanska 2002 [53]**	Investigation of MSD prevalance, risk factors, and treatment among Polish dentists	**Population**	Dentists	**Male/female**	10.8%/89.2%	12-months	56.3%	-	-	60.1%	37.3%	25.4%	44.0%	47.8%			-
		**N-participant**	268	**Age (year, mean ± SD)**	n/a												
		**Response rate**	n/a	**Country**	Poland												
**Adams et al., 2013 [32]**	Investigation of MSD prevalence in gynecologic surgeons in United States	**Population**	Gynecologists	**Male/female**	50.3%/49.7%	12-months	72.9%	61.6%	-	75.6%	66.6%	-	60.9%	-	-	-	-
		**N-participant**	495	**Age (year, mean ± SD)**	47												
		**Response rate**	7.90%	**Country**	United States												
**Knudsen et al., 2014 [44]**	Investigation of MSD prevalence and risk factors among resident orthopaedic surgeons in United States	**Population**	Orthopedists	**Male/female**	75%/25%	12-months	59.4%	35.5%	-	54.8%	34.4%	3.1%	19.4%	9.7%	22.6%	22.6%	-
		**N-participant**	32	**Age (year, mean ± SD)**	29.5 ± 2.5												
		**Response rate**	82.00%	**Country**	United States												
**Liang et al., 2012 [6]**	Investigation of MSD prevalence and role of ergonomics among dermatologists in United States	**Population**	Dermatologists	**Male/female**	71%/29%	12-months	65.2%	53.3%	-	63.1%	61.5%	13.8%	36.9%	-	24.8%	20.5%	-
		**N-participant**	354	**Age (year, mean ± SD)**	44.5 ± 9.0												
		**Response rate**	43.00%	**Country**	United States												
**Szeto et al.. 2009 [52]**	Investigation of MSD prevalence and physical and psychosocial factors among general surgeons in Hong Kong	**Population**	Surgeons	**Male/female**	82.2%/17.8%	12-months	82.9%	52.6%	-	68.1%	57.8%	-	-	-	-	-	-
		**N-participant**	135	**Age (year, mean ± SD)**	35.2												
		**Response rate**	27.00%	**Country**	Hong Kong												
**Okuyucu et al., 2017 [4]**	Investigation of MSD prevalencecharacteristics, and severity among amongst obstetrics and gynaecology practitioners in United Kingdom	**Population**	Obstetrics and gynaecology trainees	**Male/female**	n/a	12-months	8.0%	30.0%			18.0%	18.0%		13.0%			-
		**N-participant**	59	**Age (year, mean ± SD)**	32.7												
		**Response rate**	76.00%	**Country**	United Kingdom												
**Okuyucu et al., 2019 [47]**	Investigatation of MSD prevalence, severity and psychosocial risk factors among midwives in United Kingdom	**Population**	Midwives	**Male/female**	3.5%/96.5%	12-months	45.3%	29.5%	-	71.4%	44.5%	12.3%	25.6%	28.9%	31.8%	22.9%	-
		**N-participant**	630	**Age (year, mean ± SD)**	42.76 ± 11.4												
		**Response rate**	n/a	**Country**	United Kingdom												
**McLeod et al., 2017 [46]**	Investigation of MSD prevalence, risk factors and treatment among Australian osteopaths	**Population**	Osteopaths	**Male/female**	38.7%/61.3%	12-months	6.7%	12.2%	-	13.3%	11.1%	12.2%	41.1%	2.2%	1.1%	-	-
		**N-participant**	160	**Age (year, mean ± SD)**	36.4												
		**Response rate**	9.00%	**Country**	Australia												

**Table 3:** Overall MSD prevalence by healthcare profession.

Overall MSD Prevalence by Healthcare Profession											
Dentists		Midwives		Nurses		Osteopaths		Physiotherapists		Surgeons	
Anton et al. [5]	93.0%	Okuyucu et al. [4]	90.0%	Anap et al. [9]	81.0%	McLeod et al. [46]	58.0%	Adegoke et al. [33]	91.3%	Liang et al. [6]	90.0%
Kierklo et al. [43]	92.0%	Okuyucu et al. [47]	92.0%	Asghari et al. [36]	92.5%			Alrowayeh et al. [34]	47.6%	Szeto et al. [52]	83.0%
Leggat and Smith [45]	87.2%			Choobineh et al. [39]	84.4%			Campo et al. [38]	28.0%		
				Kee and Seo [18]	56.8%			Chung et al. [10]	92.4%		
				Munabi et al. [3]	80.8%			Cromie et al. [17]	91.0%		
				Pugh et al. [48]	75.8%			Glover et al. [8]	68.0%		
				Ribeiro et al. [50]	89.0%			Jang et al. [41]	71.4%		
				Smith et al. [51]	70.0%			Muaidi and Shanb [22]	47.7%		
				Tinubu et al. [23]	84.4%			Vieira et al. [54]	96.0%		

**Table 4:** MSD prevalence by body area and healthcare profession, summarized by continent.

		Main Body Area									
		Neck	Upper Back	Mid Back	Lower Back	Shoulders	Elbows/Forearms	Wrists/Hands/Fingers	Hips/Thighs	Knees	Ankles/Feet
Physiotherapists	Africa *	31.1%	14.3%	-	69.8%	22.2%	5.6%	20.6%	6.3%	15.9%	9.5%
	Asia	25.2%	19,0%	39.8%	21.6%	12.7%	23.2%	12.9%	20.5%	22.2%	7.5%
	America	33.0%	2.4%	66,0%	6.6%	19.1%	21.7%	10.2%	19.2%	2.1%	12.6%
	Oceania *	47.6%	-	62.5%	-	41.0%	22.9%	13.2%	21.8%	33.6%	7.3%
	Europe	33.5%	49.8%	37.2%	62.9%	33.5%	25.7%	24.4%	25.2%	30.4%	19.1%
Nurses	Africa	32.5%	26.3%	-	53,0%	22.6%	11.3%	22.7%	15.7%	29.8%	24.2%
	Asia	30.3%	27.4%	5.0%	50.4%	31.9%	9.5%	24.6%	17.8%	34.9%	32.6%
	America	-	-	-	-	-	-	-	-	-	-
	Oceania *	35.5%	11.3%	-	53.2%	32.3%	3.2%	16.1%	9.7%	17.7%	11.3%
	Europe *	50.1%	40.9%	-	63.1%	37.8%	7.2%	28.4%	8.9%	25.2%	26.4%
Dentists	Africa	-	-	-	-	-	-	-	-	-	-
	Asia	62.5%	82,0%	-	64.0%	26.0%	23.0%	42.0%	-	-	-
	America *	68.5%	67.4%	-	56.8%	60.0%	21.1%	69.5%	19.0%	13.7%	15.8%
	Oceania	60.3%	35.2%	-	56.2%	48.9%	11.7%	33.6%	13.2%	22.0%	12.4%
	Europe	51.7%	20,0%	-	46.5%	28.7%	20.3%	36.6%	35.6%	31.9%	31.4%
Surgeons	Asia *	82.9%	52.6%	-	68.1%	57.8%	-	-	-	-	-
	America	70.1%	50.8%	-	65.4%	55.1%	8.5%	39.1%	9.7%	23.7%	21.6%

Midwives and osteopaths are not included in this table due to the small number of studies. *: indicates the continents for which only one study was available.

## Data Availability

Not applicable.

## Appendix A

**Table A1:** Detailed quality appraisal of the 36 articles included in review using the modified CONSORT 2010 checklist [29].

			Introduction		Methods											Results						Discussion				Total	
		Title and Abstract	Background and Objective		Trial Design		Participants	Intervention	Outcomes		Sample Size		Randomizatoin and Sequence Generation		Statistical Methods	Participant Flow	Baseline Data	Numbers Analyzed	Outcomes and Estimation	Ancillary Analyses	Harms	Limitations	Generalizability	Interpretation	Funding	Score/19	% Present Criteria
		1	2A	2B	3A	3B	4	5	6A	6B	7A	7B	8A	8B	9	10	11	12	13	14	15	16	17	18	18		
PT	**Adegoke et al. 2008 [33]**	1	1	1	0	-	1	1	1	-	0	-	0	-	1	0	1	1	0	0	-	1	0	0	0	**10**	**53%**
PT	**Alrowayeh et al. 2010 [34]**	1	1	1	0	-	1	1	1	-	0	-	0	-	1	0	1	1	0	0	-	1	1	1	0	**12**	**63%**
PT	**Anyfantis and Biska 2017 [35]**	1	1	1	0	-	1	1	1	-	0	-	0	-	1	0	0.5	1	1	1	-	0	0.5	0.5	0	**10**	**61%**
PT	**Campo et al. 2008 [38]**	1	1	1	0	-	1	1	1	-	0	-	0	-	1	0	0.5	1	1	1	-	1	1	1	0	**13**	**71%**
PT	**Chung et al. 2013 [10]**	1	1	1	0	-	1	1	1	-	0	-	0	-	1	1	1	1	0	0	-	0	1	0.5	0	**11**	**61%**
PT	**Cromie et al. 2000 [17]**	1	1	1	0	-	1	1	1	-	0	-	1	-	1	1	1	1	1	0.5	-	1	1	1	0	**15**	**82%**
PT	**Glover et al. 2005 [8]**	1	1	1	0	-	1	1	1	-	0	-	1	-	1	1	1	1	1	0.5	-	1	1	1	1	**16**	**87%**
PT	**Jang et al. 2006 [41]**	1	1	1	0	-	1	1	1	-	0	-	1	-	1	1	1	1	1	0	-	1	1	0.5	1	**15**	**82%**
PT	**Muaidi and Shanb 2016 [22]**	1	1	1	0	-	1	1	1	-	0	-	0	-	1	1	1	1	0	0.5	-	0	1	0.5	0	**11**	**63%**
PT	**Vieira et al. 2015 [54]**	1	1	1	0	-	1	1	1	-	0	-	0	-	0.5	1	1	1	0	0.5	-	1	1	0.5	1	**12**	**71%**
Nurse	**Pugh et al. 2020 [48]**	1	1	1	0	-	1	1	1	-	0	-	0	-	1	1	1	1	0	0.5	-	1	1	1	1	**14**	**76%**
Nurse	**Ribeiro et al. 2016 [50]**	1	1	1	0	-	1	1	1	-	0	-	0	-	1	1	1	1	0	0.5	-	1	0.5	1	0	**12**	**68%**
Nurse	**Asghari et al. 2019 [36]**	1	1	1	0	-	1	1	1	-	1	-	1	-	1	1	1	1	1	1	-	0.5	1	1	0	**16**	**87%**
Nurse	**Kee and Seo 2007 [42]**	1	1	1	0	-	1	1	1	-	0	-	0	-	1	1	1	1	0	0	-	0	0.5	0.5	0	**10**	**58%**
Nurse	**Tinubu et al. 2010 [23]**	1	1	1	0	-	1	1	1	-	0	-	0	-	1	1	1	1	1	1	-	1	0.5	0.5	0	**13**	**74%**
Nurse	**Munabi et al. 2014 [3]**	1	1	1	0	-	1	1	1	-	1	-	0	-	1	1	1	1	1	0	-	0.5	1	1	0	**14**	**76%**
Nurse	**Choobineh et al. 2006 [39]**	1	1	1	0	-	1	1	1	-	0	-	1	-	1	1	1	1	1	1	-	0	0.5	0.5	0	**13**	**74%**
Nurse	**Smith et al. 2004 [51]**	1	1	1	0	-	1	1	1	-	0	-	0	-	1	1	1	1	1	1	-	1	1	1	0	**15**	**79%**
Nurse	**Anap et al. 2013 [9]**	1	1	1	0	-	1	1	1	-	0	-	0	-	0.5	1	1	0.5	0	0	-	0	0.5	0.5	0	**8**	**53%**
Nurse	**Yeung et al. 2005 [55]**	1	1	1	0	-	1	1	1	-	0	-	0	-	1	1	0.5	1	0	1	-	1	0.5	0.5	0	**11**	**66%**
Nurse	**Attar et al. 2014 [55]**	1	1	1	0	-	1	1	1	-	1	-	1	-	1	1	1	1	1	1	-	1	1	1	0	**17**	**89%**
Dentist	**Rabiei et al. 2011 [49]**	1	1	1	0	-	1	1	1	-	0	-	0	-	1	1	1	0.5	1	0	-	0	0.5	1	0	**11**	**63%**
Dentist	**Hayes et al. 2009 [40]**	1	1	1	0	-	1	1	1	-	0	-	0	-	1	1	1	1	1	1	-	0.5	0.5	1	0	**13**	**74%**
Dentist	**Khan and Yee Chew 2013 [42]**	1	1	1	0	-	1	1	1	-	0	-	0	-	1	1	1	1	1	0.5	-	1	1	1	0	**14**	**76%**
Dentist	**Kierklo et al. 2011 [43]**	1	1	1	0	-	1	1	1	-	0	-	0	-	1	1	0	1	0	1	-	0	0.5	0.5	0	**10**	**58%**
Dentist	**Ayers et al. 2009 [37]**	1	1	1	0	-	1	1	1	-	0	-	1	-	1	1	1	1	0	1	-	0	1	0.5	1	**14**	**76%**
Dentist	**Leggat et al. 2006 [45]**	1	1	1	0	-	1	1	1	-	0	-	1	-	1	1	1	1	0	0.5	-	0.5	0.5	0.5	0	**11**	**68%**
Dentist	**Szymanska 2002 [53]**	1	1	1	0	-	0.5	1	1	-	0	-	0	-	1	1	0	0.5	0	1	-	0	0.5	0.5	0	**8**	**53%**
Dentist	**Anton et al. 2002 [5]**	1	1	1	0	-	0.5	1	1	-	0	-	0	-	1	1	1	1	1	1	-	1	0.5	0.5	0	**12**	**71%**
Surgeron	**Liang et al. 2012 [6]**	1	1	1	0	-	1	1	1	-	0	-	0	-	1	1	1	0.5	0	0.5	-	1	0.5	0.5	0	**10**	**63%**
Surgeron	**Adams et al. 2013 [32]**	1	1	1	0	-	1	1	1	-	0	-	0	-	1	1	1	1	1	1	-	1	0.5	0.5	1	**14**	**79%**
Surgeron	**Knudsen et al. 2014 [44]**	1	1	1	0	-	1	1	1	-	0	-	0	-	0.5	1	1	1	0	0	-	1	0.5	0.5	1	**11**	**66%**
Surgeron	**Szeto et al. 2009 [52]**	1	1	1	0	-	1	1	1	-	0	-	0	-	1	1	1	0.5	1	1	-	1	0.5	0.5	1	**13**	**76%**
Midwife	**Okuyucu et al. 2017 [4]**	1	1	1	0	-	1	1	1	-	0	-	0	-	0	0.5	1	1	0	0	-	1	0.5	0.5	0	**9**	**55%**
Midwife	**Okuyucu et al. 2019 [47]**	1	1	1	0	-	1	1	1	-	0	-	0	-	1	1	1	1	1	1	-	0	0.5	0.5	0	**12**	**68%**
Osteopath	**McLeod et al. 2017 [46]**	1	1	1	0	-	1	1	1	-	0	-	0	-	1	1	1	1	1	0.5	-	1	0.5	1	0	**13**	**74%**

PT = Physiotherapists/Physical therapists; Rating code: 1: the criterion is present; 0.5: the criterion is partially present; 0: the criterion is absent. Columns 3B, 6B, 7B, 8B, and 15 were not filled in because the criteria in the list were difficult to apply to the articles included in the review.

**Table A2:** Job risk factors identified for all healthcare professionals (percentage of sample and corresponding number of participants in brackets.

Job Risk Factor—Activity That Causes Injury	Anap et al. [9]	Tinubu et al. [23]	McLeod et al. [46]	Adegoke et al. [33]	Anyfantis and Biska [35]	Chung et al. [10]	Glover et al. [8]	Muaidi and Shanb [25]	Liang et al. [6]	Szeto et al. [52]
Profession	Nurses	Nurses	Osteopaths	PT	PT	PT	PT	PT	Surgeons	Surgeons
Number of responders	N = 228	N = 118	N = 160	N = 120	N = 252	N = 157	N = 2688	N = 690	N = 354	N = 135
Number of responders with work-related MSD	**N = 203**	**N = 100**	**N = 93**	**N = 114–115**	**N = 252**	**N = 157**	**N = 1515–1648**	**N = 690**	**N = 354**	**N = 135**
Bending or twisting forward	48.5% (98)	45.8% (46)	3.3% (3)	62.6% (72)	-	77.7% (122)	56% (893)	61.3% (423)	-	-
Lifting or transferring dependent patients	52.4% (106)	50.8% (51)	5.5% (5)	67.8% (78)	-	80.3% (126)	56% (908)	48.8% (337)	-	-
Working in the same position for a long time	47.6% (97)	55.1% (55)	8.8% (8)	71.3% (82)	-	73.2% (115)	67% (1085)	87.8% (606)	-	88.9% (120)
Treating a large number of patients in 1 day	41% (83)	44.9% (45)	-	83.5% (96)	-	90.4% (142)	67% (1081)	68.7% (474)	-	-
Performing the same task over and over	22.4% (45)	14.4% (14)	53% (49)	52.2% (60)	90% (227)	86.6% (136)	73% (1203)	74.4% (513)	-	37.8% (51)
Working in an awkward/cramped position	35.2% (71)	33.1% (33)	-	64.6% (73)	70% (176)	81.5% (128)	44% (691)	72.3% (499)	-	88.9% (120)
Performing manual therapy techniques	32.7% (66)	40% (40)	23% (21)	67.8% (78)	-	72.0% (113)	49% (777)	69.3% (478)	-	-
Unanticipated sudden movement or fall by patient	21.8% (44)	28.8% (29)	-	40.9% (47)	-	66.9% (105)	39% (618)	42.0% (290)	-	-
Carrying or moving heavy materials/equipment/patient	42.4% (86)	42.4% (42)	-	55.7% (64)	-	64.3% (101)	30% (464)	42.0% (290)	-	-
Working when physically fatigued/in an injured state	29.6% (60)	32.2% (32)	38.2% (36)	52.2% (60)	-	77.7% (122)	52% (823)	72.6% (501)	58% (205)	-
Assisting patients with gait activities	17.2% (35)	12.7% (13)	-	35.7% (41)	-	61.1% (96)	37% (582)	72.3% (499)	-	-
Lack of rest breaks during the day	31.4% (64)	39% (39)	-	61.7% (71)	-	89.8% (141)	41% (644)	44.4% (306)	76% (269)	-
Working at or near physical limits	20.3% (41)	23.7% (24)	-	46.9% (54)	-	64.3% (101)	44% (686)	53.5% (369)	-	-
Working with confused or agitated patients	10% (20)	16% (16)	-	28.9% (33)	-	62.4% (98)	25% (384)	42.0% (290)	-	-
Inappropriate training in injury prevention	12.1% (25)	27.1% (27)	-	29.6% (34)	-	42.7% (67)	14% (212)	38.8% (268)	-	-
Reaching or working away from your body	41.2% (84)	31.6% (32)	-	17.4% (20)	-	56.1% (88)	51% (800)	55.8% (385)	-	-
Work scheduling (overtime, irregular shifts, length of workday)	26% (53)	33.9% (34)	43% (40)	3.5% (5)	-	62.4% (98)	18% (271)	47.4% (327)	-	-
Forceful exertion	-	-	-	-	-	-	-	-	-	44.4% (60)

**Table A3:** Response and treatment used to reduce symptoms of MSD identified for all healthcare professionals (percentage of sample and corresponding number of participants in brackets).

	Okuyucu et al. [4]	Anap et al. [9]	Tinubu et al. [23]	McLeod et al. [46]	Chung et al. [10]	Muaidi and Shanb [22]	Cromie et al. [17]	Glover et al. [8]	Anyfantis and Biska [35]	Adegoke et al. [33]	Liang et al. [6]
Profession	Midwives	Nurses	Nurses	Osteopaths	PT	PT	PT	PT	PT	PT	Surgeons
Number of responders	N = 59	N = 228	N = 118	N = 160	N = 157	N = 690	N = 536	N = 2688	N = 252	N = 120	N = 354
Number of responders with work-related MSD	**N = 50**	**N = 203**	**N = 100**	**N = 93**	**N = 157**	**N = 690**	**N = 298–512**	**N = 1100–1825**	**N = 252**	**N = 115**	**N = 354**
Medication	62% (31)	-	-	-	-	-	-	-	-	-	22% (78)
Visiting a physician	38% (19)	-	-	-	-	-	-	39% (705)	33% (83)	-	-
Be rested due to injury	-	-	-	33.7% (31)	-	-	-	32% (580)	-	-	-
Officially reported the injury	-	-	-	-	-	-	-	16% (286)	-	-	-
Alteration of working habits	-	-	-	-	-	-	-	9% (152)	-	-	-
Limitation of contact time with patients	-	-	-	28.2% (26)	-	-	-	10% (169)	3% (8)	-	-
Considering changing their job	-	-	-	9% (8)	-	-	-	1% (11)	-	-	-
Modified treatment technique	-	-	-	-	-	-	-	59% (1057)	12% (30)	-	-
Sought PT formally or informally from a colleague	-	-	-	-	-	-	-	61% (1087)	-	-	-
I get someone else to help me handle a heavy patient	-	57.1% (116)	50.4% (50)	-	-	64.2% (443)	86.9% (352)	-	-	76.5% (88)	-
I use physical therapist assistants to perform physically stressful tasks	-	-	-	-	-	-	32.9% (98)	-	-	-	-
I modify patient’s position/my position	-	41.2% (84)	40.3% (40)	40.4% (38)	51.6% (81)	91.9% (634)	98.2% (503)	8% (146)	25% (63)	91.3% (165)	-
I use other body part in order to perform manual treatment/nurse procedure	-	19.2% (39)	20.2% (20)	-	51.6% (81)	94.6% (653)	80.9% (372)	-	-	50.4% (58)	-
I adjust plinth/bed height prior to the treatment of a patient	-	18.3% (37)	21.8% (22)	-	47.1% (74)	97.8% (675)	95.4% (455)	-	-	69.5% (80)	-
I select techniques/procedure that will not cause or aggravate discomfort	-	23.2% (47)	30.5% (30)	60.7% (56)	30.6% (48)	98.2% (678)	77.4% (366)	-	15% (38)	80% (92)	-
I warm up and stretch before performing my work manual techniques, nurse duties	-	16.2% (33)	32.8% (33)	-	14.0% (22)	68.2% (471)	20.5% (96)	-	-	28.7% (33)	-
I get someone else to help me handle a heavy patient	-	-	-	-	13.4% (21)	67.5% (528)	-	-	-	-	-
I pause regularly so I can stretch and change posture	-	10.2% (21)	14.3% (14)	27% (25)	7.0% (11)	84.2% (581)	78.0% (393)	-	10% (25)	75.7% (87)	-
I stop a treatment if it causes or aggravates my discomfort	-	28.1% (57)	33.6% (34)	-	7.0% (11)	82.8% (571)	71.9% (343)	-	-	67.5% (78)	-
I use electrical therapy instead of manual therapy to avoid stressing an injury	-	-	-	-	5.7% (9)	-	24.5% (96)	-	-	48.7% (56)	-
I modify my nursing procedure in order to avoid stressing an injury	-	50.2% (102)	45.4% (45)	-	-	-	-	-	-	-	-
